# RETRACTED: Alhakamy et al. Encapsulation of Lovastatin in Zein Nanoparticles Exhibits Enhanced Apoptotic Activity in HepG2 Cells. *Int. J. Mol. Sci.* 2019, *20*, 5788

**DOI:** 10.3390/ijms26115263

**Published:** 2025-05-30

**Authors:** Nabil A. Alhakamy, Osama A.A. Ahmed, Hibah M. Aldawsari, Mohammad Y. Alfaifi, Basma G. Eid, Ashraf B. Abdel-Naim, Usama A. Fahmy

**Affiliations:** 1 Department of Pharmaceutics, Faculty of Pharmacy, King Abdulaziz University, Jeddah 21589, Saudi Arabia; aldosarih@gmail.com (H.M.A.); usamafahmy@hotmail.com (U.A.F.); 2 Department of Pharmaceutics & Industrial Pharmacy, Faculty of Pharmacy, Minia University, Minia 61519, Egypt; 3 Biology Department, Faculty of Science, King Khalid University, Abha 9004, Saudi Arabia; alfaifi@kku.edu.sa; 4 Department of Pharmacology and Toxicology, Faculty of Pharmacy, King Abdulaziz University, Jeddah 21589, Saudi Arabia; beid@kau.edu.sa (B.G.E.); abnaim@yahoo.com (A.B.A.-N.)

The journal retracts the article titled “Encapsulation of Lovastatin in Zein Nanoparticles Exhibits Enhanced Apoptotic Activity in HepG2 Cells” [1], cited above.

Following publication, concerns were brought to the attention of the Editorial Office regarding a range of image irregularities contained within this article [1] and image duplication between articles [1,2] published by the same author group.

Adhering to our standard procedure, the Editorial Office and Editorial Board conducted an investigation that confirmed image inaccuracies in Figure 4 and duplication between Figures 6A,B and 7A,B in [1], and Figures 5A,B and 6A,B in [2]. Following discussions, the authors collaborated but were unable to satisfactorily explain these irregularities, or provide appropriate raw material for Editorial Board evaluation. Consequently, the Editorial Board has lost confidence in the reliability of the findings and has decided to retract this publication [1], as per MDPI’s retraction policy (https://www.mdpi.com/ethics#_bookmark30).

This retraction was approved by the Editor-in-Chief of the *Int. J. Mol. Sci.*

The authors did not provide a comment on this decision.
